# Opportunities for Low-Barrier HIV Testing in the U.S. Deep South: Findings from a Survey in Alabama

**DOI:** 10.1007/s10461-025-04910-9

**Published:** 2025-11-13

**Authors:** Lynn T. Matthews, Madeline C. Pratt, Michael Murphy, DeAndra Tuyishime, Shericka Williams, Katherine Waldon, Tara Wood, Ashley Tarrant, Bretia Gordon, Dustin M. Long, Sonya Heath, Ingrid V. Bassett, Mirjam-Colette Kempf

**Affiliations:** 1 Division of Infectious Diseases, University of Alabama at Birmingham, Heersink School of Medicine, 1720 2nd Avenue South, ZRB 212, Birmingham, AL 35294 USA; 2 Division of Infectious Diseases, Yale University School of Medicine, New Haven, CT, United States of America; 3 University of Alabama at Birmingham, Heersink School of Medicine, Birmingham, AL, United States; 4 Five Horizons Health Services, Southern Region, Montgomery, AL USA; 5 Five Horizons Health Services, Tuscaloosa, AL USA; 6 Alabama Regional Medical Services, Birmingham, AL USA; 7 Medical Advocacy and Outreach, Montgomery, AL USA; 8 School of Medicine, Wake Forest University, Winston-Salem, NC USA; 9 Department of Medicine, Massachusetts General Hospital, Boston, MA USA; 10 School of Nursing, University of Alabama at Birmingham, Birmingham, AL USA

**Keywords:** HIV testing, HIV, Rural health, Alabama, EHE, Low barrier care

## Abstract

The HIV epidemic remains a critical public health challenge in the Southern U.S., where barriers such as healthcare shortages, structural racism, and social determinants of health exacerbate disparities. Mobile- and self-HIV testing offer promising approaches to address these barriers. Partnering with a community-based healthcare organization in Alabama, we surveyed 181 individuals accessing facility- and community-based HIV testing from August 2022 to November 2023. Most participants were Black (92%), women (78%), and heterosexual (83%), with 73% reporting annual incomes below $50,000. While 72% felt unlikely to acquire HIV, a fifth reported forgoing healthcare due to competing financial needs like housing (23%) and food (21%). Barriers to care were rated as "very slight" to "somewhat of a problem," and medical mistrust was highest in group disparities (mean 27.2/60). Community-level HIV stigma averaged 3.45/5, while social support scored 61.1/100. Mobile-based HIV testing was favorably rated for acceptability (3.9/5), appropriateness (4/5), and feasibility (4/5). Among participants, 61% were aware of HIV self-testing, 21% had used it, and 47% expressed interest in future use. Findings reveal low perceived HIV risk and significant community stigma, though individual stigma was less of a barrier than factors like fear of a positive result and lack of knowledge about care. This study highlights the need for tailored interventions to improve HIV testing access for vulnerable populations across Alabama and other rural U.S. settings.

## Introduction

The HIV epidemic remains a significant public health challenge, particularly in the Southeastern United States (U.S.). Low HIV testing and diagnosis rates contribute to continued HIV transmission [1]. While confidential and free facility-based HIV testing services are available throughout the Southeastern U.S., large numbers of people remain unaware of their HIV-serostatus [2, 3]. In Alabama, an estimated 38% of adults have ever tested for HIV [4]. Challenges to HIV testing are multi-faceted, including limited access in rural areas, and social and economic determinants of health, such as historical and current structural racism, medical mistrust, poverty, and healthcare shortages. In addition, low perceived risk, confidentiality concerns, and client dissatisfaction with HIV testing services contribute to poor testing uptake [5].

Mobile HIV counseling and testing can serve as one strategy to increase testing coverage and engagement in HIV care and prevention. This “low barrier care” approach may reduce transportation barriers, mitigate facility-based stigmas, and enhance opportunities to reach prioritized populations in their communities. Mobile based HIV counseling and testing is a common strategy to promote testing in many sub-Saharan African countries with generalized HIV epidemics and other settings with health systems more focused on public health [6]. In a meta-analysis of 14 studies with >74,000 clients mostly in sub-Saharan Africa, uptake of mobile HIV counseling and testing (HCT) was 87% [7]. Limited research has explored feasibility and acceptability of these approaches in rural America, particularly in the Southeastern U.S. where HIV incidence rates remain high [8]. One study conducted in Alabama and Mississippi surveyed Black men who have sex with men (MSM) who preferred facility-based testing with a provider [9]. Some data suggest that mobile-based testing has greater reach compared to facility-based testing among young adults [7]. In studies exploring opportunities to optimize HIV testing coverage with community members/consumers, community-based strategies were identified as important to increasing HIV testing in the Southeastern U.S. [10, 11].

In addition to mobile counseling and testing, HIV self-testing (HIVST) has emerged as another important HIV testing modality when aiming to reach underserved key populations. A review of randomized controlled trials reporting on self-testing outcomes from 2006 to 2019 found that HIVST increased testing uptake by 1.45 times and increased the mean number of HIV tests (primarily among MSM) by more than 2.5 times compared to facility-based testing [12]. However, downsides to HIVST have been its association with poorer linkage to care rates compared to facility-based testing [12].

To address low testing rates in rural Alabama, we utilized public and private testing data to identify zip codes with lower testing coverage [13]. We then shared maps illustrating low HIV testing rates with community-based AIDS service organizations who provide testing outreach and worked with those partners to distribute surveys to clients accessing HIV testing via both facility- and community-based testing. Here we describe demographics, social and behavioral characteristics that may impact testing uptake for this population of people accessing HIV testing through our community partners in Alabama. We describe their self-reported acceptability of HIV self-testing, and feasibility/acceptability of mobile based testing.

## Methods

### Study Population and Setting

We partnered with a community-based AIDS service organization covering 28 counties in Alabama’s “Black-belt” to describe the populations accessing mobile-based and community outreach testing services, aiming to explore acceptability and feasibility of mobile-based and HIV-self testing approaches. For this study we included adults (ages 18–65) living in Alabama and participating in HIV testing and counseling services provided by the community partner.

### Community Partnership

The community partners for this project included Medical Advocacy and Outreach (MAO) and the Black Belt Community Foundation (BBCF). MAO was an AIDS service organization in Montgomery, AL, that provided HIV and STI testing, prevention, and treatment services, with satellite offices located in rural counties in southern AL. In 2022, due to financial challenges at MAO, Five Horizons Health Services assumed care of all clients within MAO's service area. Five Horizons Health Services, a nonprofit community-based organization that provides services across southeastern Alabama, through the Alabama Black Belt, and across East Mississippi. They also chose to continue this research project. BBCF is a community organization working to organize philanthropic funding for the Black Belt region to promote health, prosperity, inclusivity, education, and creativity [14].

MAO, in partnership with the Alabama Department of Public Health, planned to roll out mobile-based HIV counseling and testing using a new mobile clinic van in 2021. The first goal of this academic and community partnership was to utilize data to inform mobile testing outreach conducted by MAO. Previously published research explored preferences for receiving mobile HIV testing services, including locations, advertising, and messaging [5] and examined HIV testing coverage and time from diagnosis to viral suppression across AL by ZIP code [13]. As a continuation of this work, the aim of this study was to evaluate implementation of a mobile testing outreach program implemented by MAO, using quantitative surveys of those accessing services via the mobile unit.

Study aims were adjusted as unforeseen circumstances arose, such as the COVID-19 pandemic resulting in delays in delivery of and outfitting of the mobile testing van. Additionally, our partner organization underwent internal leadership changes over the course of 2021 and 2022. We shifted our strategy from solely focusing on mobile van testing and counseling to instead focus on any form of outreach testing (including for non-communicable diseases) that occurred outside of the clinic.

### Recruitment

Participants were recruited from clinical and outreach testing sites, including the main Five Horizons Southern Region clinical location, college and university campuses, health fairs, job resource centers, substance use support centers, and churches. After participating in HIV testing or counseling, patients were provided with a study recruitment flyer that allowed them to scan a QR code linking directly to the survey. Information about the study, participant rights, and consent were provided, and participants consented electronically before entering the questionnaire. Some participants chose to complete the survey at the testing site, while others completed the survey after leaving the site.

Participants received $30 for completing the survey, which was expected to take 15–30 min to complete.

### Survey

The survey included demographic questions about race, employment, household income, education, gender, and sexuality. Questions about mobile HIV counseling and testing experiences asked about time spent accessing the mobile van, completing HIV testing, waiting for results, and receiving counseling, as well as implementation measures related to acceptability, feasibility, and appropriateness of mobile testing services [15]. Additional questions assessed current HIV-serostatus and perceived likelihood of contracting HIV. Participants were asked about their prior use of HIV self-testing or home-testing kits and whether they were interested in utilizing or distributing these kits in the future. Further questions examined barriers to accessing healthcare and competing needs [16, 17], HIV-related stigma [18–21], medical mistrust [22], and social support [23], informed by the Levesque model for healthcare access [24].

### Ethical Approval and Informed Consent

This study was reviewed and approved by the Institutional Review Board at the University of Alabama at Birmingham (IRB#-300007605) and the Research Review Committee at Medical Advocacy and Outreach and Five Horizons.

### Statistical Analysis

Descriptive statistics were calculated for all continuous variables (mean and standard deviation) and categorical variables (frequency and percent). All analyses were conducted using SAS 9.4 (SAS Institute Inc., Cary, NC, USA).

## Results

### Demographics

**Fig. 1:**
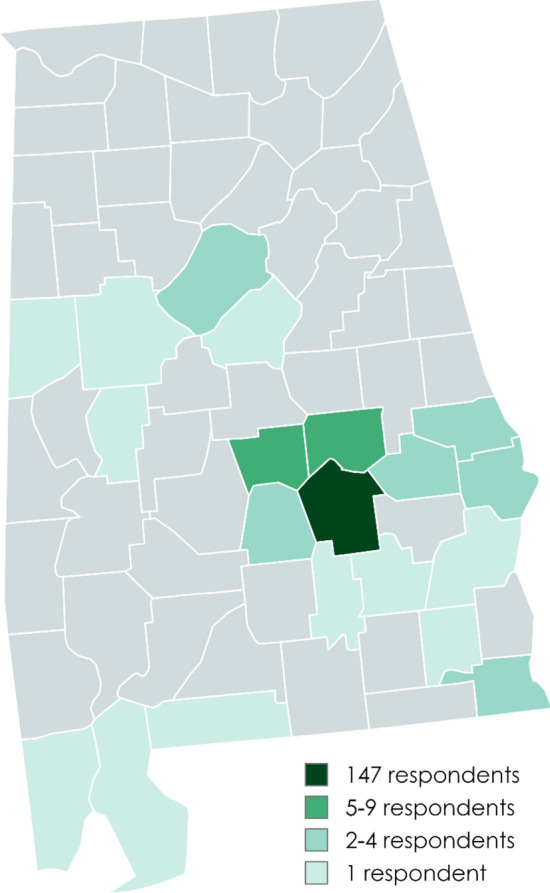
Counties of residence of participants, all within the U.S. state of Alabama

197 surveys were completed between August 11, 2022, and November 16, 2023. After removing duplicates (based on a combination of address, phone number, and date of birth), there were 189 unique survey responses. Mean (SD) age was 36.6 (11.4) years, and the vast majority (N = 147, 77.8%) of participants resided in Montgomery County (Fig. 1). Most survey participants were Black (N = 173, 91.5%) and neither Hispanic nor Latino (N = 182, 96.3%). Most were women (N = 148, 78.3%), identified as heterosexual/straight (N = 156, 82.5%), employed fulltime (N = 123, 65.1%), and earning an annual household income below $50,000 (N = 137, 72.5%). Thirty-six percent (N = 68) of participants had completed an associate’s degree or higher education. Most (N = 128, 68%) reported low personal HIV risk perception (Table 1).

**Table 1:** Demographics for N = 189 survey participants

N = 189	N (%)
Age (years), mean (SD)	36.8 (11.6)
Race	
Black or African American	173 (92%)
White	11 (6%)
American Indian or Alaska Native	2 (1%)
Ethnicity	
Hispanic or Latino	5 (3%)
Gender	
Cis-gender man	35 (19%)
Cis-gender woman	148 (78%)
Gender diverse*	6 (4%)
Employment	
Full-time	123 (65%)
Part-time	27 (14%)
Unemployed	15 (8%)
Full-time student	13 (7%)
Education	
Primary school, no diploma	8 (4%)
High school graduate, or GED	56 (30%)
Some college	55 (29%)
College graduate	48 (35%)
Post-graduate studies	20 (11%)
Sexual orientation	
Heterosexual or straight	156 (83%)
Gay, Queer, Homosexual, or Same Gender Loving	20 (11%)
Bisexual	9 (5%)
HIV-serostatus—Person without HIV	179 (95%)
HIV risk perception^a^	
Not likely	128 (72%)
Somewhat likely	36 (20%)
Very likely	4 (2%)
Extremely likely	0 (0%)

*Transgender, gender non-binary, asexual, none of the above

^a^Self-reported 4-point Likert measure of perception asked as, “What is your gut feeling about how likely you are to be infected with HIV?”

### Social Factors

Our survey assessed prevalence of going without health care because of other competing needs. Most (N = 134, 71%) of respondents reported no competing needs. About a fifth reported going without healthcare in the last 6 months because funds were needed for housing (N = 42, 22%), food (N = 39, 21%), and/or clothing (N = 30, 16%) (Table 2).

**Table 2:** Survey findings: acceptability, appropriateness, and feasibility of HIV testing among N = 189 participants

N = 189	N (%) Mean (SD)
Competing needs^a^	
Went without health care because money was needed for food	39 (21%)
Went without health care because money was needed for clothing	30 (16%)
Went without health care because money was needed for housing	42 (23%)
Went without food because money was needed for health care	17 (9%)
Went without clothing because money was needed for health care	18 (10%)
Went without housing because money was needed for health care	16 (9%)
HIV stigma^b^	3.45 (0.9)
Group based medical mistrust^c^	27.2 (5.9)
Suspicion	2.55 (0.9)
Group disparities	3.28 (1.11)
Lack of support from healthcare providers	2.62 (0.74)
Barriers to care scale^d^	2.48 (1.3)
*Missing N* = *7*	
Barriers affecting participants’ personal decision to test for HIV^e^	2.7 (0.9)
*Missing N* = *8*	
Cost (or lack of re-imbursement by insurance)	2.56 (1.5)
Not knowing where to receive specialty care for HIV	2.54 (1.4)
Not feeling at risk for HIV infection	2.64 (1.4)
Concern that HIV testing will reflect badly on me as a person	2.46 (1.4)
Concern of being judged by my health care provider	2.46 (1.4)
Afraid of test results	2.54 (1.5)
Concern about others finding out that I got tested for HIV	2.36 (1.4)
Afraid of testing procedures	2.29 (1.5)
Social support^f^	61.1 (22.6)
*Missing N* = *9*	

^a^Dichotomous yes/no questions in the form of “In the last 6 months, have you ever had to go without [variable] because the money was needed for [variable]?” [16]

^b^10 statements about the treatment of people with HIV in society and by health care workers. 5-point Likert scale ranging from 1 to 5 (1 = “strongly disagree” to 5 = “strongly agree”). Overall score is an average of the individual item scores, ranging from 1 to 5 with 5 being the greatest level of stigma. [21]

^c^Medical mistrust was assessed using a 12-item scale that measures mistrust of health care systems, professionals, and treatment provided to individuals of the respondent's ethnic or racial group. The response key was a Likert-type scale ranging from 1 (strongly disagree) to 5 (strongly agree) and the score range was 12–60, with a higher score indicating more medical mistrust [22]

^d^Barriers to Care Scale (BACS): 4-point Likert scale with 8 questions across 4 domains: geography/distance, medical and psychological, community stigma, personal resources. Individual items were scored 1–4 (1 = “no problem at all” to 4 = “major problem”), and final scores were calculated by summing the scale’s 8 items and dividing by 8, producing a score ranging from 1 to 4, with higher numbers indicating higher overall problem severity [17]

^e^5-point Likert scale with 8 questions. Participants asked to rank how each factor affects their decision to get tested for HIV (1 = “not at all” to 5 = “completely”). Overall score is the sum of the score across all 8 questions divided by 8, producing a score ranging from 1 to 5, with 5 indicating the strongest barriers [43]

^f^Multi-dimensional social support including emotional/informational, tangible, affection, and positive social interaction. Each item was rated on a 5-point scale ranging from *not true at all* (0) to *true nearly all of the time* (4). A total score was obtained by summing responses to all items, with higher scores reflecting greater availability of support. Range 0–100 with higher score indicating more social support [44]

We assessed barriers to care [16] with a mean (SD) score of 2.5 (1.3) indicating most participants describe barriers to care as “very slight” to “somewhat of a problem”. When assessing barriers to HIV testing we observed minimal variation across potential barriers with scores between a mean of 2–3 indicating these barriers were selected as “very slight” to “somewhat of a problem” for most (Table 2).

Group-based medical mistrust [22] was assessed with a score of 27.2 (5.9) with a possible range of 12–60. Within the medical mistrust sub-categories of suspicion, group disparities, and lack of support from healthcare providers, the highest score (indicating most mistrust) was in group disparities (e.g. in most hospitals, people of different ethnic groups receive the same kind of care).

We assessed community-level HIV stigma with average response of 3.45 (0.9) out of a possible 1 (lowest stigma) to 5 (most stigma). Participants reported a mean social support score of 61.1 (22.6) out of 0–100 with 100 representing the most social support.

### Mobile Based HIV Testing and Counseling

While all participants experienced some form of non-facility-based healthcare in order to be enrolled, 41 (22%) reported prior HIV counseling and testing in a non-facility setting. Among this group, most (N = 32, 78%) reported being tested between 11 am and 5 pm and most (N = 32, 80.6%) reported waiting <10 to 20 min to test and most (N = 28, 68%) spent 10–20 min with a counselor.

We included 9 items exploring acceptability, appropriateness, feasibility of mobile-based testing. All were scored on a 5-point scale with 5 indicating most favorable ratings with acceptability 3.9 (0.9), appropriateness 4 (0.9), and feasibility 4 (0.9). Table 3 shows the proportion responding agree/strongly agree to the specific items.

**Table 3:** Survey findings: acceptability, appropriateness, and feasibility of HIV testing among N = 181 participants

N = 181, *missing* = *8*	Agree or Strongly Agree N, %
*Acceptability*	
Mobile-based HIV testing meets my approval	126 (66.7%)
Mobile-based HIV testing is appealing to me	130 (68.8%)
I like mobile-based HIV testing	134 (70.9%)
I welcome mobile-based HIV testing	144 (76.2%)
I would recommend mobile-based HIV testing to a friend	146 (77.3%)
I would like to use mobile-based HIV testing again	115 (60.9%)
*Appropriateness*	
Mobile-based HIV testing seems fitting to increase HIV testing	144 (76.2%)
Mobile-based HIV testing seems suitable for testing people in my community	145 (76.7%)
*Feasibility*	
Mobile-based HIV testing seems easy to use	145 (76.7%)

### HIV Self-Testing

**Fig. 2:**
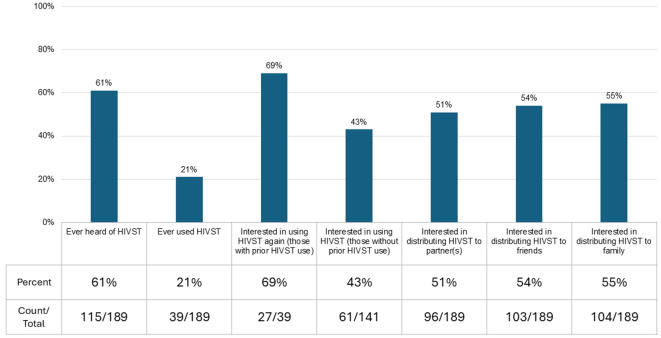
Proportion of participants who had heard of, used, and were interested in HIVST kits

Most (N = 115, 61%) respondents had heard of HIVST and 39 (21%) had used HIVST kits. Most (N = 27, 69%) of this subset reported interest in using an HIVST kit again. Among all respondents, 88 (47%) reported interest in using an HIVST kit and 17 (9%) were not sure. Common responses for not wanting to use an HIVST included preference to be with a counselor at the time of testing (N = 51, 27%) and “I have unanswered questions” (N = 10, 5%). About half of respondents expressed interest in distribution of HIVST kits to partners (N = 96, 51%), friends (N = 103, 54%), and family (N = 104, 55%) (Fig. 2).

## Discussion

In this cross-sectional survey among 181 people in Alabama with prior experience accessing non-facility-based healthcare, we found that mobile-based HIV counseling and testing was acceptable to approximately two thirds of participants, and HIV self-testing was acceptable (i.e. something they would be interested in pursuing) among ~50% of the survey participants. Among those unsure whether to access self-testing services, participants indicated that they preferred a trusted healthcare professional to be part of their testing experience. These findings suggest an opportunity to reach more people by diversifying HIV testing approaches offered through HIV testing community outreach services in the South.

There are very limited data regarding client perceptions or experiences with mobile-based testing in rural America. One group published on their experience conducting pop-up HIV screening and linkage to PrEP in Washington D.C. with high uptake and acceptability [25], while data published from Alabama regarding facility based testing preferences indicated that sexual/gender and/or racial minority men prefer facility based testing [9] vs mobile-based or self-testing [8]. Indeed, a recent review highlights the importance of diverse choices based on varied preferences [26]. Higher acceptability of non-facility-based options in our cohort may reflect prior experience and/or demographics of our cohort which was older, Black women, who largely did not view themselves at risk for HIV. In the global HIVST literature, reaching women with HIVST who then distribute kits and instructions to men (who face additional barriers to accessing HIV testing and care [27, 28]) has been successful [29, 30].

While mobile based testing and counseling was acceptable and feasible to clients, it requires investment in systems and infrastructure to implement [31]. Comparisons of the costs per person tested using mobile- versus clinic-based testing are not widely available and are especially limited for U.S. settings. One estimate from East Africa found that costs vary from US $10 to $35 for mobile strategies [32], with higher costs per person testing positive in areas with lower HIV prevalence. In rural South Africa, a cost-effectiveness analysis of home- versus clinic-based HIV counseling and testing found that home testing costs US $29 compared to $38 per person for clinic testing [33]. In our prior work, clients and key stakeholders shared that bundling HIV prevention services with other services (e.g., vaccinations) and screening tests (e.g. for non-communicable diseases such as hypertension, diabetes, cancer) is an effective strategy [5, 11]. Working with funders and implementers to diversify services offered through mobile units so that HIV testing/care is not the only (stigmatized) service is critical and complicated given how many HIV services are funded through distinct mechanisms that do not allow for additional services being offered.

More research has explored HIVST in the U.S. Many of our participants had experienced HIVST and almost half of those were interested in accessing HIVST again. The most common reasons for not wanting to use HIVST (again) included wanting to be with a provider or having additional questions, highlighting the importance of supporting people to complete testing, linking those who test negative to preventative care, and those who test positive to HIV care. Tran and colleagues recently reviewed 173 studies of HIVST support approaches including in-person referrals, written referrals, phone help, visual guides, as well as digital app supports—the most common were in-person and printed support materials [34]. Most of the pioneering work in this space continues to come from countries with high HIV prevalence, robust public health systems, and large rural populations, such as South Africa. Adapting interventions to support and link people accessing HIVST to prevention services and treatment is important to successfully reach populations in underserved areas in the U.S.

Findings from this study show that even among those who seek HIV testing, perceptions of being vulnerable to HIV are low. HIV stigma is perceived to be widespread in the community, yet respondents rated individual anticipated stigma as less of a barrier to HIV testing than lack of risk perception, costs of testing and receiving treatment, lack of knowledge on where to receive specialty care, and personal fear of a positive test result. Further, medical mistrust among our sample of primarily Black female participants in the South was similar to scores among Latino men from California [35] but lower than scores among Black men in New York [36]. Another study found that Black women in Connecticut experience higher levels of medical mistrust and lower comfort seeking HIV prevention from providers than white women [37]. A South Carolina study exploring HIV knowledge, risk perceptions, and testing behaviors among college students found similar lack of risk perception with less than 20% of respondents perceiving themselves to be at-risk for acquiring HIV and only 8% ever having been tested [38]. Findings from a survey of health care providers in the U.S. South confirm widespread provider misconceptions about HIV risk, barriers to care, and stigmatization [39].

### Strengths/Limitations

Limitations of our study include a small sample size of people who were accessing HIV testing services, which may not reflect the views of people who have not yet tested for HIV. Due to challenges with mobile based testing roll out by our community partners, we did not limit recruitment to people accessing mobile based testing. Thus, some participants did not have any experience with mobile based testing and or HIVST so their responses were more hypothetical. The demographics of the population who participated are more representative of middle- and older-aged adults and mostly cis-gender Black women, and therefore may not be as reflective of other populations. However, given that women of color in the Southeastern U.S. are highly vulnerable to HIV acquisition, understanding the needs of this population is important. Our findings are, however, unique and provide insights into how to improve HIV testing reach to help end the rural HIV epidemic in the U.S.

### Lessons Learned and Next Steps

This work informs ongoing projects across Alabama to improve access to HIV testing among vulnerable populations. Our community partner team has now fully launched a mobile HIV counseling and testing unit with extensive outreach across the state [31]. In addition, members of this team are funded to improve HIV testing outreach as part of a multi-component intervention to improve the HIV care cascade in coastal Alabama [40]. Our state department of health (Alabama Department of Health) implemented a partnership to deploy home-based HIV and STI testing to increase opportunities for people in Alabama to know their HIV-serostatus using well-supported home-based testing through commercial entities [41]. Dr. Matthews’ team has subsequent funding to partner with this program to enroll women vulnerable to HIV into a longitudinal cohort [42]. This work and this suite of research projects have highlighted the challenges of implementing low-barrier testing programs. As a result, our investigators are partnering with community testing entities to organize regular gatherings of people leading this work within AIDS service organizations and public and academic medical center health entities to share lessons learned, brainstorm about new opportunities, and continue to grow the work together.
